# Impact of *Helicobacter pylori* Eradication Therapy on Platelet Counts in Patients With Chronic Idiopathic Thrombocytopenic Purpura

**DOI:** 10.5539/gjhs.v8n7p35

**Published:** 2015-11-03

**Authors:** Mohamadreza Amiri

**Affiliations:** 1 University of Medical Sciences, Department of Pediatrics, Zahedan, Iran

**Keywords:** ^13^C-urea breath test, chronic idiopathic (Autoimmune) thrombocytopenic purpura, eradication therapy, *Helicobacter pylori*, platelet count, *Helicobacter pylori* stool antigen test

## Abstract

This study was a before and after clinical evaluation of *Helicobacter pylori* eradication on platelet counts in a group of 23 patients with chronic Idiopathic (Autoimmune) thrombocytopenic purpura (CITP). *H. pylori* infection was identified in patients by a ^13^C-urea breath test and confirmed by an *H. pylori* stool antigen test. Eradication was conducted in patients testing positive. Infected (*n* = 10) and uninfected (*n* = 13) patient groups did not differ with respect to age, gender, history of previous splenectomy, treatment with anti-D, current treatment with corticosteroids, or initial platelet counts. *H. pylori* eradication was successful in eight infected CITP patients, with two patients not responsive to treatment. Compared to the uninfected group, patients in the infected group who responded to eradication therapy had significantly increased platelet counts after six months (56.2 ± 22.2 *vs*. 233 ± 85.6 ×10^3^ million cells/L; *P* < 0.01), whereas platelet counts in the non-responding patients and uninfected group did not differ after this period of time. *H. pylori* eradication promotes significant platelet count improvement in patients with CITP. Thus, all patients with CITP should be tested and treated for *H. pylori* infections.

## 1. Introduction

Idiopathic (Autoimmune) thrombocytopenic purpura is a condition in which the immune system destroys platelets, thus impairing normal blood clotting. The condition typically occurs one to four weeks after exposure to common viral infections and is associated with petechiae and purpura symptoms in 1–4-year-old children who were previously healthy (Paul Scott, Montgomery, Blanchard, & Czinn, 2007). In almost 20% of patients, this disorder is present for extended periods (more than 12 months), and is therefore described as chronic Idiopathic thrombocytopenic purpura (CITP). Treatment for CITP involves control of symptoms and prevention of serious bleeding (Lanzkowsky, 2005). Splenectomy is effective in 64–88% of CITP-infected children, with intravenous anti-D and immunoglobulins, corticosteroids, and rituximab as other treatment options (Paul Scott et al., 2007).

*Helicobacter pylori* is a gram-negative bacterium that represents one of the most common infections in children and affects approximately half of the world population (Ciancarelli et al., 2002). It is most commonly transmitted by fecal-oral or oral-oral routes (Lanzkowsky, 2005), and causes chronic inflammation in the stomach, the amount of which varies by strain and from host to host. Although there are no clinical consequences to *H. pylori*-induced gastritis, the final outcome from infection can be life threatening. *H. pylori* is also known to cause duodenal ulcer, gastric ulcers, adenocarcinoma of the distal stomach, and gastric mucosa-associated lymphoid tissue lymphoma (Rowland et al., 2004). Interestingly, platelet counts is often increased in CITP patients treated for *Helicbacter pylori* infection (Paul Scott et al., 2007).

In a study of patients with a resistant form of CITP, Azarm and Khami found that a majority of patients were infected with *H. pylori*, and those who received eradication therapy experienced a significant increase in platelet counts (Azarm & Khami, 2005). Similarly, bacterial eradication therapy increased platelet counts nearly eight-fold in a subset of CITP patients with *H. pylori* infection in a study by Ferrara et al. (2009) However, some reports failed to show recovered platelet counts in patients successfully treated for *H. pylori*, and thus screening for infection was not recommended in children (Veres et al., 2009). The purpose of the present study was to assess the effect of *H. pylori* eradication on platelet counts in CITP patients.

## 2. Method and Materials

### 2.1 Patient Selection

This study was a before and after clinical evaluation of patients from the Pediatric Hematology Clinic of Medical Sciences University (Zahedan, Iran) that received treatment for CITP between 2010 and 2011. Patients were excluded from the study for: i) previous history of *H. pylori* eradication; ii) presence of other serious diseases such as malignant tumors, or cardiovascular, renal or liver diseases; iii) treatment with > 0.5 mg/kg/day prednisolone during or one month prior to the study; iv) need for platelet injection and/or other medicines that increase platelet count; v) lack of cooperation with study requirements.

All of the patients included in the study were diagnosed with CITP in 2010–2011 by bone marrow aspiration and treated with isolated thrombocytopenia for more than 12 months without any detectable cause involved. At first a total of 24 patients were studied, however, one of them died due to a brain hemorrhage and was excluded from the study. None of the other patients had life-threatening bleeding 12 months prior to the study or after.

### 2.2 Testing for H. pylori Infection

*H. pylori* infection was assessed using the ^13^C-urea breath test (^13^C-UBT). Proton pump inhibitors and antibiotics were not prescribed for four weeks prior to the test. The ^13^C-UBT was conducted after an overnight fast, and breath samples were collected before and 30 min after ingestion of ^13^C-urea (Helico State; Simac Diagnostica, Veenendal, Netherlands) dissolved in 100 mL of water. Doses of ^13^C-urea were 50 mg in children younger than 6 years of age, and 75 mg in children over 6 years. ^13^C available in expiratory CO_2_ was measured via an isotope radio mass spectrometric method (Heli View; Medichems, Korea), and values higher than 4% were considered as positive (Muccio & Jackson, 2009). Testing for *H. pylori* infection was confirmed by a positive result from an *H. pylori* stool antigen test (HP Ag T Kit; Genesis Diagnosis, Cambridge, UK), which demonstrates 92% sensitivity and 96% specificity (Gisbert & Pajares, 2001).

### 2.3 H. pylori Eradication

*H. pylori*-infected patients were treated with a triple eradication therapy comprised of 15 mg/kg/day clarithromycin and 20 mg/kg/day metronidazole (bid) for two weeks, and a proton pump inhibitor (omeperazole; 1 mg/kg/day, bid) for one month (Paul Scott et al., 2007). After 4–6 weeks, a ^13^C-UBT was performed to confirm successful eradication. Two patients were resistant to triple eradication therapy, and were then treated with a two-week quadruple therapy: omeperazole (1 mg/kg/day, bid), metronidazole (20 mg/kg/day, bid), bismuth subcitrate (two 250 mg tablets, q8h), and clarithromycin (15 mg/kg/day, bid) (Rowland et al., 2004). These patients remained positive for *H. pylori* infection, and were therefore described as non-responsive.

### 2.4 Platelet Quantification

At the beginning of the study, 2 cc venous blood samples were collected from each patient. Samples were diluted 1:100 in ammonium oxalate and platelet counts were measured. Platelet counts were evaluated each month for up to six months after eradication treatment.

### 2.5 Statistical Analysis

Data were analyzed using Student’s *t*, Mann-Whitney U (if not normally distributed), Kolmogorov–Smirnov, and *χ*^2^ tests. Central and dispersion indices are to describe the quantitative data, and relative and absolute frequencies are used to describe the qualitative data. *P* < 0.05 was considered as a statistically significant difference for all of the tests.

## 3. Results

**Figure 1:**
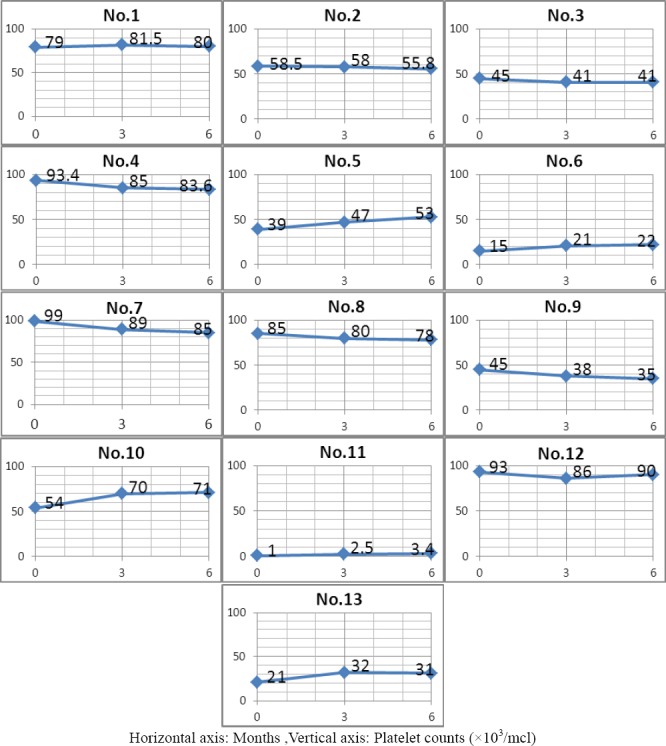
Platelet counts over the six-month study period for all uninfected patients

**Figure 2:**
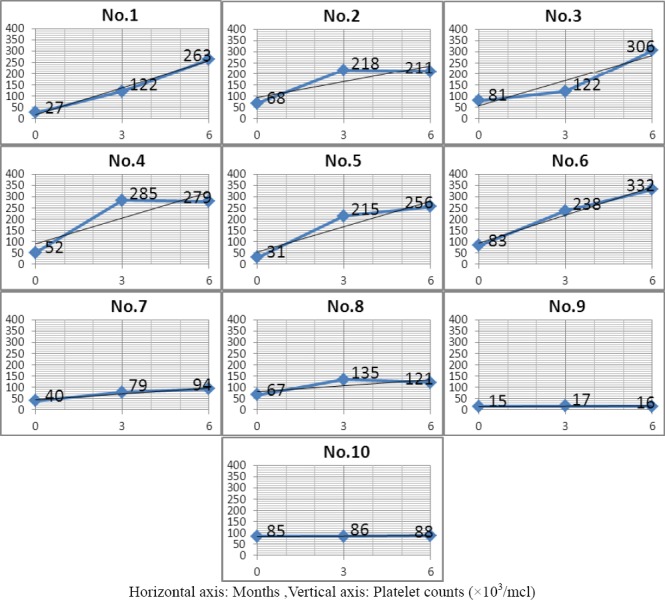
Platelet counts over the six-month study period for all patients infected with Helicobacter pylori

Twenty-four (14 male and 10 female) patients were diagnosed with CITP and were under treatment in the Pediatric Hematology Clinic during 2010–2011. One of the male patients died as a result of cerebral hemorrhage and was excluded from the study. Of the 23 patients included in the study, ten (43.5%) were infected with *H. pylori*. The mean age of the infected group (*n*= 10) was not significantly different from the uninfected group (*n* = 13) (9.5 ± 4.7 *vs*. 10.2 ± 5.0 y). Furthermore, there were no significant differences regarding sex, previous history of splenectomy, initial platelet count, anti-D treatment or corticosteroid therapy. Of the ten patients treated with *H. pylori* eradication therapy, only eight showed successful elimination of the pathogen upon subsequent evaluation (treatment responders). Initial platelet counts in the responders and non-responders did not differ from the uninfected group (Table 1). However, there were differences in platelet counts six months after eradication therapy. Specifically, only the group successfully treated for *H. pylori* infection showed a significant increase in platelet count (*P* < 0.01). The platelet count after treatment in this group was increased by 177 ± 82 ×10^3^/million cells/L (mcl), compared with a change of 62 ± 20 mcl in the uninfected group. Although platelet counts increased by 2.0 ± 1.3 ×10^3^/mcl in the non-responders, this was not significantly different from initial values or from the uninfected group. Platelet counts over the six-month follow up for all patients are presented in Figure (1, 2).

**Table 1:** Platelet counts

	Uninfected (*n* = 13)	Treatment responders (*n* = 8)	Treatment non-responders (*n* = 2)
Initial platelet count, ×10^3^ mcl	56.0 ± 32.2	56.2 ± 22.2	50.0 ± 49.5
Final platelet count, ×10^3^ mcl	56.1 ± 27.8	233.0 ± 85.6	52.0 ± 50.9
*P*	0.98	< 0.01	0.28

Abbreviations: mcl, million cells per liter.

## 4. Discussion

In recent years, several studies reported HP prevalence and the effect of eradication therapy on platelet counts improvement in patients with chronic ITP. In this study, platelet counts change was studied in a group of 23 patients with chronic ITP who referred to the pediatric hematology clinic of University of Medical Science for treatment with the diagnosis of the respective illness during 2010-2011.

For convenience, the patients were divided into three groups:

- HP-infected patients with chronic ITP who were treated successfully
- HP-infected patients with chronic ITP who were not treated successfully
- HP-uninfected patients with chronic ITP who were not treated for eradication therapy

10 patients were infected with HP and eradication therapy was successful in eight of them. (80%).

Both HP-infected and HP-uninfected patients were followed up for six months (there was no significant difference between two groups in terms of age, gender, treatment with anti-D, initial platelet counts at the baseline, history of previous splenectomy and current treatment with corticosteroids).

Platelet counts (CBC Diff) was performed once a month. After six months, there was a significant increase in platelet counts in 8 patients infected with HP who were treated successfully. However, there wasn’t any platelet counts increase in patients who weren’t treated successfully (2 patients) as well as a group of 13 patients uninfected with HP so they were not treated. The results of this study demonstrate that successful eradication of H. pylori significantly increases platelet counts in Iranian CITP pediatric patients.

Prevalence of H.pylori infection in this study was 43.5% and in studies performed by other researchers the prevalence of HP infection in patients with chronic ITP was reported differently. It seems that different economic and social conditions dominated on the studied societies were the cause of the discrepancy. On the other hand, the present study was performed on the age group below 20 years while other studies were performed on different age groups (including age range of adults in addition to children).

The results of this research are consistent with the results obtained by some researchers including:

1). Study performed by Ferrara et al. (2009) in Department of Pediatrics, University of Naples, Italy. They studied 24 children with chronic ITP; 8 were infected with HP (prevalence rate=33.3%). Successful eradication therapy was performed in 6 cases accompanied by significant increase in platelet counts.

2). Study performed by Ryugo Sato et al.. performed in Oita University, Japan. They studied 53 patients, of them 39 were infected with HP (prevalence rate=73%). 32 patients were treated by eradication therapy which was successful in 27 patients accompanied with significant increase in platelet counts (Sato et al., 2004).

On the other hand, the result of the present study is inconsistent with the results of some studies including:

1). Study performed by Veres et al. (2009) performed in Children’s Medical Center, Budapest, Hungary. They studied 27 children with chronic ITP, of them, 2 patients were infected with HP (prevalence rate=7%). The two children were treated successfully by HP eradication therapy; however; there wasn’t any improvement in their platelet counts in 10 month follow up.

2). Study performed by Treepongkaruna et al., (2009) published in a paper entitled ’’lack of improvement in platelet count after HP eradication therapy in children with chronic ITP’’. Of 55 children with chronic ITP, 16 cases were infected with HP (prevalence rate=29%) and eradication therapy was performed successfully in treatment group (7 patients); however, there was no significant difference between treatment and control groups in platelet counts after 6 months.

As seen in Figure 2, in patients number 1,3,5,6 and 7 (HP-infected patients) there was gradual monthly increase in platelet counts after eradication therapy which was continued up to six months. In patients number 2, 4 and 8 platelet counts increased up to three months after eradication therapy and it remained relatively stable up to the end of the sixth months. In patients 9 and 10 who didn’t respond to HP eradication therapy, there wasn’t any increase in platelet counts.

A limitation of this and previous studies is the relatively short follow-up period. It is possible that these effects may not persist when examined over longer periods. Another important limitation is that the number of study subjects was limited. Further studies with larger patient populations are therefore required to confirm the beneficial effect of H. pylori eradication on platelet counts.

However, in this study HP eradication therapy induced improvement of platelet counts in 8 of 10 HP-infected patients with chronic ITP and they indicated acceptable and significant response after six months eradication therapy.

There are many hypotheses on the mechanism by which HP can cause chronic ITP. One of them is molecular similarity mechanism between certain antigens present on the microbe and some of platelet glycoproteins; HP induces the production of antibodies against the respective antigens and makes platelets to be trapped and demolished in the reticuloendothelial and spleen by interaction mechanism. The possible role of some variants of HP which are positive in terms of CagA has been raised in this regard. Lewis antigens are also raised as one of target antigens. These antigens are expressed by some HP variants and absorbed by platelets can be a target for anti-Lewis antibodies in patients with favorable genetic background (Stasi & Provan, 2008).

## 5. Conclusion

Although the mechanism of thrombocytopenia in CITP is unclear, eradication therapy may be an effective method to increase platelet counts. Therefore, it is recommended that all patients with CITP should be evaluated for H. pylori infection and receive treatment if needed.
